# Hemophagocytic lymphohistiocytosis in the context of hepatitis-associated severe aplastic anemia: A case report

**DOI:** 10.1097/MD.0000000000045430

**Published:** 2025-10-31

**Authors:** Jianli Xu, Hailong Yuan, Gang Chen, Jianhua Qu, Huan Wang, Rong Chen, Ming Jiang

**Affiliations:** a Hematologic Disease Center, the First Affiliated Hospital of Xinjiang Medical University, Urumqi, Xinjiang, China; b Xinjiang Uygur Autonomous Region Research Institute of Hematology, Urumqi, Xinjiang, China.

**Keywords:** aplastic anemia, hemophagocytic syndrome, lymphoproliferative disorder, severe aplastic anemia, transplantation

## Abstract

**Rationale:**

Hemophagocytic lymphohistiocytosis (HLH) is a severe immune dysregulation syndrome. Hepatitis-associated severe aplastic anemia (SAA) is a specific subtype of acquired aplastic anemia characterized by concomitant hepatitis, leading to a challenging diagnosis and poor prognosis. Here, we present a rare case of HLH in the context of hepatitis-associated SAA, emphasizing the need for early recognition and individualized management.

**Patient concerns:**

The patient, a 19-year-old male, presented with recurrent fever, jaundice, and cytopenias, indicative of hepatitis.

**Diagnoses:**

Bone marrow aspiration and biopsy suggested SAA, while peripheral blood next-generation sequencing detected *Candida tropicalis* infection on 2 separate occasions. Despite combination antifungal therapy with amphotericin B and posaconazole, the bilirubin level continued to rise. Additional tests revealed an elevated soluble CD25 level of 3659 U/mL, decreased natural killer cell activity of 1.8%, and a serum ferritin level > 2000 μg/L. The final diagnosis was HLH.

**Interventions:**

Dexamethasone and ruxolitinib were administered to control the fever. Subsequently, the patient underwent HLA-matched allogeneic bone marrow and peripheral blood hematopoietic stem cell transplantation for SAA.

**Outcomes:**

The patient’s symptoms, including fever and fatigue, resolved.

**Lessons:**

This case highlights that HLH should be considered in SAA patients with unexplained fever, poor response to anti-infectives, and elevated bilirubin. A treatment strategy addressing both bone marrow failure and hyperinflammation is essential. In this patient, allogeneic hematopoietic stem cell transplantation served as a successful curative intervention by targeting the shared pathophysiology of HLH and SAA. However, given the inherent limitations of a single case, the generalizability of our findings and the efficacy of allogeneic hematopoietic stem cell transplantation require validation through larger, controlled studies.

## 1. Introduction

Hemophagocytic lymphohistiocytosis (HLH), also known as hemophagocytic syndrome, is an immune disorder caused by genetic defects, malignant tumors, infections, autoimmune diseases, etc, leading to systemic inflammation and multi-organ failure, with a high mortality rate. The diagnostic criteria for adult HLH are based on the HLH-2004 criteria for children.^[1]^ HLH can be diagnosed if gene mutations are detected or if 5 or more of the following 8 criteria are met: fever lasting more than 1 week, peak fever > 38.5°C; splenomegaly; cytopenia in 2 or more blood cell lineages (hemoglobin < 90 g/L, platelet count < 100 × 10^9^/L, absolute neutrophil count < 1.0 × 10^9^/L); hypertriglyceridemia (>3 mmol/L) and/or hypofibrinogenemia (<1.5 g/L); hemophagocytosis in bone marrow, spleen, liver, or lymph nodes; serum ferritin ≥ 500 μg/L; decreased or absent natural killer (NK) cell activity; and elevated soluble CD25.^[2]^ In adults, infection and malignant tumors are the most common causes of HLH, with lymphoma being the most prevalent malignancy.^[3]^ Aplastic anemia (AA) is associated with increased apoptosis of bone marrow stem cells and can be classified into congenital AA and acquired AA based on etiology. Hepatitis-associated AA, a specific type of acquired AA occurring concurrently or subsequently with hepatitis, is characterized by jaundice, pancytopenia, and hypocellular bone marrow. Most cases of hepatitis-associated AA are severe aplastic anemia (SAA) with a poor prognosis.^[2]^ The incidence of AA-related HLH is low, and reports are limited.^[4,5]^ Here, we report a case of HLH in the context of hepatitis-associated SAA, aiming to enhance understanding of such diseases and provide treatment references.

## 2. Case report

**Figure 1.**
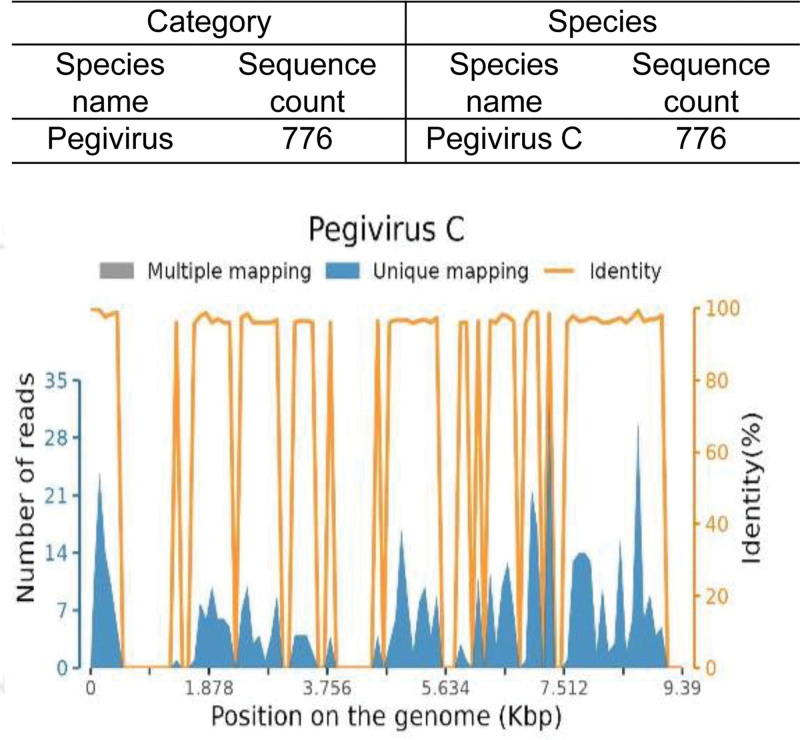
RNA-next-generation sequencing results reported infection with pegivirus C with a count of 776.

**Figure 2.**
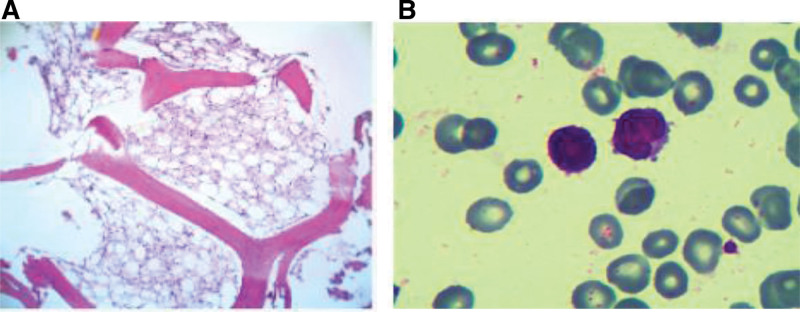
Bone marrow analysis. (A) Results of bone marrow biopsy of right posterior superior iliac spine (H&E staining, ×40 magnification), demonstrating reduced proliferation in all hematopoietic lineages, a lower granulocytic/erythroid ratio, decreased granulocytes, and sporadically distributed megakaryocytes. Interstitial adipose tissue increased, with no phagocytosis noted. (B) Results of bone marrow cytology of the right posterior superior iliac spine. (Wright-Giemsa staining, ×400 magnification), indicating a reduction in the proliferation of nuclear cells and both granulocytic and erythroid lineages, along with an increased ratio of mature lymphocytes. No hemophagocytosis was observed.

The patient was a 19-year-old male who initially presented to a local hospital in June 2023 with skin and mucosal jaundice. He was diagnosed with acute jaundiced hepatitis and discharged after receiving hepatoprotective and corticosteroid therapy. Following discharge, the steroid dose was tapered and discontinued after bilirubin and transaminase levels normalized. Approximately 3 days after discontinuing medication, the patient developed petechiae on both lower limbs. He was re-admitted 1 week after the appearance of petechiae and 5 days after the onset of fever (peak temperature 39°C). Blood tests revealed thrombocytopenia. A bone marrow puncture raised the possibility of AA. For further management, the patient was transferred to our department from the emergency center with a provisional diagnosis of pancytopenia. On admission, the patient presented with fever, pancytopenia, and ecchymosis on the limbs. He also reported severe fatigue, profound weakness, and a persistent high fever that significantly impaired his daily functioning. Routine blood tests showed a white blood cell count of 0.26 × 10^9^/L, absolute neutrophil count of 0.02 × 10^9^/L, hemoglobin of 69 g/L, and platelet count of 5 × 10^9^/L (Table 1). Biochemical tests revealed aspartate aminotransferase of 17.96 U/L, alanine aminotransferase of 25.0 U/L, lactate dehydrogenase of 118.46 U/L, total bilirubin of 39.46 umol/L, direct bilirubin of 19.27 umol/L, and triglycerides of 0.61 mmol/L (Table 1). The coagulation test showed that prothrombin time was 11.1 seconds, fibrinogen concentration was 3.53 g/L, and D-dimer concentration was 290.0 ng/mL (Table 1). The concentration of C-reactive protein, interleukin-6, and procalcitonin was 36.9 mg/L, 12.30 pg/mL, and 0.36 ng/mL, respectively (Table 1). Real-time quantitative PCR analysis of peripheral blood revealed that the Epstein-Barr virus (EBV)-DNA and cytomegalovirus (CMV)-DNA were both below the lowest detectable value. Blood culture indicated *Candida tropicalis* infection. Metagenomic next-generation sequencing for hepatitis A, B, C, and E showed negative results. RNA-next-generation sequencing results reported infection with human pegivirus C with a count of 776 (Fig. 1). Metagenomic next-generation sequencing results for EBV and CMV were negative. Other microbiological and serological tests, as well as anti-nuclear antibody tests, were negative. Chest CT revealed a slight pulmonary fibro stripe in the left upper lobe of the lung, without pleural effusion. Abdominal ultrasound did not show hepatosplenomegaly. Superficial lymph nodes were not enlarged. The CD4^+^/CD8^+^ ratio was 1.0 (Table 1). The bone marrow biopsy demonstrated reduced proliferation in all hematopoietic lineages, a lower granulocytic/erythroid ratio, decreased granulocytes, and sporadically distributed megakaryocytes (Fig. 2A). Additionally, there was an increase in interstitial adipose tissue with no phagocytosis noted. On the other hand, the bone marrow cytology indicated a reduction in the proliferation of nuclear cells and granulocytic and erythroid lineages, along with an increased ratio of mature lymphocytes (Fig. 2B). The overall morphology was normal with no megakaryocytes or evidence of phagocytosis. The chromosomal karyotype was 46, XY. Genetic testing results for inherited bone marrow failure syndromes were negative. Based on these results, a diagnosis of hepatitis-associated SAA was established.

**Table 1:** Laboratory parameters pre-transplantation and at 2 months post-transplantation.

Variables	Results		Normal range
	Pre-transplantation	+2 months post-plantation	
White blood cell count (×10^9^/L)	0.26	3.90	4.00–10.00
Absolute neutrophil count (×10^9^/L)	0.02	2.30	1.80–6.20
Hemoglobin (g/L)	69.00	123.00	130.00–175.00
Platelet count (×10^9^/L)	5.00	153.00	125.00–350.00
Aspartate aminotransferase (U/L)	17.96	23.00	17.00–59.00
Alanine aminotransferase (U/L)	25.00	31.00	21.00–72.00
Total bilirubin (μmol/L)	39.46	21.00	3.00–22.00
Direct bilirubin (μmol/L)	19.27	15.00	0.00–19.00
Triglycerides (mmol/L)	0.61	0.58	0.56–1.70
Prothrombin time (s)	11.10	12.50	11.00–15.00
Fibrinogen (g/L)	3.53	2.40	2.00–4.00
D-dimer (ng/mL)	290.00	156.00	0–200.00
Serum ferritin (μg/L)	>2000.00	355.00	20.00–150.00
Soluble CD25 (U/mL)	3659.00	1041.00	223.00–710.00
NK cell activity (%)	1.80	3.70	7.00–40.00
Lactate dehydrogenase (U/L)	118.46	254.00	120.00–250.00
C-reactive protein (mg/L)	36.90	25.00	0-10.00
IL-6 (pg/mL)	12.30	6.50	<7.00
Procalcitonin (ng/mL)	0.36	0.11	0–0.05
CD4^+^/CD8^+^ ratio	1.00	0.90	1.50–2.50
β-D-glucan (pg/mL)	>220.17	43.00	Negative: < 70 pg/mL Gray area/Uncertain: 70–95 pg/mL Positive: ≥ 95 pg/mL
Galactomannan index	1.95	0.31	Negative: < 0.5 Positive: ≥ 0.5

NK = natural killer.

Upon the definite diagnosis of SAA, allogeneic hematopoietic stem cell transplantation (allo-HSCT) was recommended, and HLA typing was immediately performed with the patient’s younger sister (the patient has only one sibling). During the waiting period for the HLA typing results, the patient was treated with immunosuppressive therapy involving cyclosporine A and eltrombopag to address his clinical condition effectively. Additionally, the patient was treated with meropenem, vancomycin, voriconazole, cefoperazone/sulbactam, ganciclovir, and dexamethasone (5 mg/day), but still experienced fever. HLH examinations showed: serum ferritin > 2000 μg/L, soluble CD25 at 3659 U/mL, and NK cell activity at 1.8% (Table 1). Genetic testing results for primary HLH were negative, ruling out the diagnosis of primary HLH. Following the HLH-2004 criteria, the diagnosis was HLH (related to SAA). Treatment involved dexamethasone at 10 mg/m^2^/day, but there was no significant improvement in fatigue symptoms, and the patient’s temperature continued to rise. After reducing the dexamethasone dose for 2 weeks, the patient’s temperature did not improve. Next-generation sequencing of blood samples suggested a possible diagnosis of *Candida tropicalis* candidemia, with other microbiological examination results being negative. Meanwhile, serum biomarkers of β-D-glucan (>220.17 pg/mL) and galactomannan index (1.95) were persistently elevated (Table 1), indicating fungal infection. Therefore, antifungal therapy comprising amphotericin B and posaconazole was administered. A twice-daily dose of 10 mg ruxolitinib was also used. A follow-up lung CT scan indicated some improvement in the infection. The bilirubin levels gradually decreased to 33.23 μmol/L. The patient’s body temperature initially decreased for approximately 1 week; however, the patient subsequently experienced persistent high fever, reaching temperatures of up to 40°C. Hematological findings showed pancytopenia, as evidenced by low counts in all 3 lineages (white blood cells, red blood cells, and platelets) and a severe deficiency in granulocytes. Next-generation sequencing of blood samples revealed 59 counts of *Enterobacter cloacae*, 3 counts of *Klebsiella pneumoniae*, 33,723 counts of *Candida tropicalis*, 60 counts of *Aspergillus flavus*, 5 counts of human herpesvirus 6B, and 3 counts of human herpesvirus 4. This led to further adjustments in therapy. Specifically, tigecycline was administered in combination with ceftolozane/avibactam to address bacterial infections, while amphotericin B was used in conjunction with caspofungin for antifungal treatment. The changes in therapy were made empirically based on the best available evidence in the context of limited therapeutic drug monitoring availability. Overall, the patient was suspected to have SAA, HLH, hyperbilirubinemia, liver dysfunction, and candidemia.

Meanwhile, the HLA typing results for the patient and his younger sister indicated a full HLA match, both with blood type A, Rh positive. Following this, we promptly proceeded with the allo-HSCT. Following the exclusion of contraindications, the patient underwent fludarabine + busulfan + cyclophosphamide + anti-thymocyte globulin regimen conditioning on September 19, 2023. Bone marrow and peripheral blood hematopoietic stem cells provided by the patient’s sister were infused on September 27, 28, and 29. The nucleated cell count was 16.08 × 10^8^/Kg, and the CD34^+^ count was 10.06 × 10^6^/kg. The patient experienced severe bone marrow suppression after stem cell infusion, with significant reductions in all 3 lineages (white blood cells, red blood cells, and platelets). On day + 7 post-transplantation, the patient developed increased bowel movements and dark and watery stools, suggestive of gastrointestinal bleeding. Fluid management and nutritional support were provided. Due to low platelet count, to prevent severe bleeding, symptomatic treatment with NovoSeven was administered, resulting in improvement. Proactive measures were taken to prevent rejection, infections, and support the liver and stomach, and prevent vomiting. The patient’s temperature fluctuated between 38.0°C and 39.5°C, controlled with steroids. After stem cell infusion, treatment included meropenem combined with cefoperazone/sulbactam and omadacycline for anti-inflammatory purposes. Due to intermittent fever, antibiotics were changed to meropenem combined with cefoperazone/sulbactam and moxifloxacin, and antifungal therapy was switched to isavuconazole combined with caspofungin. The combination of meropenem and cefoperazone/sulbactam was selected for broad-spectrum activity against Gram-negative bacteria, specifically targeting the high local prevalence of metallo-β-lactamase-resistant *Acinetobacter baumannii*. Fifteen days post-transplantation, blood counts began to recover, although platelet levels remained suboptimal, necessitating intermittent transfusions and oral drugs to promote hematopoiesis. Despite the recovery of white blood cells, the patient still experienced temperature fluctuations. A lung CT scan revealed a fungal infection, leading to treatment with caspofungin combined with posaconazole. Re-examination of HLH markers CD25 and NK cell activity indicated no recovery yet, prompting treatment with oral ruxolitinib. Platelet levels remained unstable, requiring intermittent transfusions and outpatient follow-ups. Therapeutic modifications were implemented empirically, guided by the best available evidence, given the limited availability of therapeutic drug monitoring. These adjustments aimed to control the spectrum of polymicrobial infections until hematopoietic recovery could be achieved through transplantation. After 3 months post-transplantation, the patient’s temperature normalized, white blood cell count was within normal range, soluble CD25 decreased to 1041 U/mL, NK cell activity improved to 3.7%, and platelet transfusions were no longer needed. With the cessation of steroids, the temperature remained normal, and the ruxolitinib was gradually tapered. At 1 year and 11 months of follow-up, the patient remains free of relapse and rejection, has successfully resumed normal activities, and reports a good quality of life. The patient continues to receive regular follow-up care. The complex clinical timeline of the patient’s presentation, diagnosis, and treatment is summarized in Table 2.

**Table 2:** Summary of clinical timeline, key findings, and interventions.

Date (2023)		Clinical event and symptoms	Key diagnostic findings	Treatment interventions
June		Admitted for skin/mucosal jaundice.	Diagnosed with acute jaundiced hepatitis.	Hepatoprotective agents and steroids. Discharged with tapering oral steroids.
Late July-Early August		Petechiae on lower limbs, fever (39°C), pancytopenia, severe fatigue, and high fever.	Low platelet count; bone marrow puncture suggested AA; Pancytopenia (WBC 0.26 × 10^9^/L, Hb 69 g/L, Plt 5 × 10^9^/L); Bone marrow confirmed SAA; Blood culture: *Candida tropicalis*.	Broad-spectrum antibiotics (e.g., meropenem, vancomycin) and antifungals (e.g., voriconazole).
Mid-Late August		Persistent high fever; suspected fungal sepsis.	HLH criteria met (e.g., ferritin > 2000 μg/L, sCD25 3659 U/mL); Elevated β-D-glucan (>220.17 pg/mL) and galactomannan (1.95); mNGS confirmed a high load of *Candida tropicalis*.	Dexamethasone (10 mg/m^2^/day); Antifungal therapy switched to amphotericin B + posaconazole; Ruxolitinib added.
September	September 19	Preparation for transplant	-	Conditioning regimen (Flu/Bu/Cy/ATG) initiated.
	September 27–29	Transplantation	-	Full-match allo-HSCT from sister.
October (~Day + 7)		Increased bowel movements and dark and watery stools.	Gastrointestinal bleeding.	Symptomatic management (fluid, nutrition, NovoSeven).
Mid-October (~Day + 15)		Temperature fluctuations.	Blood counts began to recover; Lung CT: fungal infection.	Antibiotics/antifungals adjusted; Ruxolitinib continued.
November		Gradual recovery.	WBC normalized; sCD25 decreased; NK cell activity improved.	Platelet transfusions stopped; steroids ceased.
December onward		Stable temperature, stable counts.	No relapse/rejection; good quality of life.	Immunosuppressants tapered; regular follow-up.

AA = aplastic anemia, allo-HSCT = allogeneic hematopoietic stem cell transplantation, ATG = anti-thymocyte globulin, Bu = busulfan, Cy = cyclophosphamide, Flu = fludarabine, HLH = hemophagocytic lymphohistiocytosis, mNGS = metagenomic next-generation sequencing, NK = natural killer, SAA = severe aplastic anemia.

## 3. Discussion

Due to the significant overlap in clinical features and biochemical markers between HLH and AA, they are rarely detected early. For instance, both conditions can present with bleeding, fever, pancytopenia, and splenomegaly (after infection in AA). In biochemical tests, serum ferritin levels are typically elevated in HLH, while in AA patients, iron overload can result from repeated red blood cell transfusions, making the identification of AA in HLH patients more challenging. The pathogenesis of these 2 diseases may also be similar.^[1]^ In patients with HLH, there are ineffective innate immune responses to antigens, while in AA patients, apoptosis of hematopoietic precursors leads to activation of T cells, causing a cytokine storm. Hepatomegaly with elevated transaminases can occur concurrently in HLH (histologically mimicking chronic persistent hepatitis) and AA with opportunistic infections.^[6]^ Splenomegaly, a common finding in HLH, can also be observed rarely in AA following infection with EBV. HLH is classified as either a primary or secondary type. Primary HLH is caused by genetic defects, while secondary HLH is often triggered by infections or malignant tumors. Among these, EBV-induced infections account for over half of infection-induced HLH cases.^[7]^ Furthermore, the median value of EBV-DNA when progressing to HLH is 1 × 10^4^ copies/mL.^[8]^ In this study, the patient was a young male with no family history of HLH-positive cases. The initial presentation of hepatitis followed by pancytopenia pointed strongly toward hepatitis-associated SAA, a diagnosis supported by the hypocellular bone marrow and the exclusion of congenital (inherited bone marrow failure syndromes) and other acquired causes of marrow failure. The complex infection profile identified by metagenomic next-generation sequencing, including *Candida tropicalis, Enterobacter, Klebsiella, Aspergillus*, and HHV-6B/EBV, presents a significant diagnostic challenge. It is plausible that one or more of these infections could have been the primary trigger for the HLH in this profoundly immunocompromised host. The alternative diagnosis of infection-triggered HLH or severe sepsis with HLH-like hyperinflammation was seriously considered and could not be definitively excluded. However, several factors were weighed in our clinical assessment. The hyperinflammatory state progressed despite aggressive, targeted antimicrobial and antifungal therapy. Furthermore, the temporal sequence, where the onset of hepatitis and SAA preceded the fulminant HLH presentation, suggested that the profound immune dysregulation of SAA may have created the permissive environment for both the opportunistic infections and the HLH. Therefore, while infection was a major contributory factor, we hypothesize that the underlying immune pathophysiology of SAA was the predominant driver of the HLH syndrome in this case.

Hepatitis-associated AA is primarily observed in young males, with a small proportion (1%) linked to viral hepatitis.^[9]^ Recent studies demonstrate that human pegivirus C is not hepatotropic and does not replicate in liver cells, meaning it may not have a direct causal relationship with acute or chronic hepatitis.^[10–12]^ The detection rate of human pegivirus C in healthy blood donors varies by region and age, indicating its ability to independently spread.^[13,14]^ Further research indicates a notable prevalence of human pegivirus C in patients with AA, suggesting a potential association with hematological disorders rather than liver disease.^[15–17]^ In our case, while human pegivirus C was detected, its role was likely confounding. Based on the literature and the clinical sequence, we hypothesize that human pegivirus C may have been a coincidental finding or a cofactor associated with the subsequent development of SAA, but it was improbable to be the primary causative agent of the hepatitis. The definitive cause of hepatitis remains unknown.

The dysregulation of the immune system and abnormal elevations of cytokines may form a common basis for the development of hepatitis, hepatitis-associated AA, and HLH.^[18–22]^ However, the sequence of onset among these conditions remains uncertain, complicating clinical diagnosis and treatment. In this study, we propose the following explanations for the sequential diagnosis of hepatitis, SAA, and HLH: The corticosteroid therapy the patient received for acute jaundiced hepatitis may have masked some symptoms of HLH, as they can reduce inflammation and alleviate fever. Furthermore, due to the presence of pancytopenia and fever, SAA was initially prioritized in the diagnosis. In light of ineffective treatment for SAA and the subsequent emergence of HLH manifestations, the potential for HLH was reconsidered. This experience provides valuable insights: when diagnosing patients with SAA, clinicians should consider the possibility of HLH in cases presenting with unexplained fever, suboptimal anti-inflammatory treatment, and elevated bilirubin levels to prevent delays in diagnosis and treatment.

First-line therapy for HLH prefers the HLH-1994 protocol (including etoposide and dexamethasone), while the HLH-2004 protocol introduces cyclosporine A at the beginning of treatment, reducing the early mortality rate from 27% to 19%.^[23]^ Etoposide combined with standard induction chemotherapy for lymphoma-associated HLH can significantly increase the response rate and reduce mortality.^[24]^ However, regimens based on etoposide do not enhance the prognosis of adult HLH.^[25]^ Therefore, novel non-cytotoxic drugs are needed. The JAK1/2 signaling pathway serves as the downstream pathway of IFN-γ and other receptors for inflammatory cytokines. Ruxolitinib, a JAK1/2 inhibitor, can specifically target T-cell cytotoxic effects to manage HLH.^[26]^ Clinical trials show that ruxolitinib as a first-line treatment achieves a complete remission rate of 73.1% in children with HLH^[27]^ and a total response rate of 77% in adults with HLH.^[28]^ When ruxolitinib is combined with doxorubicin-etoposide-methylprednisolone, the total response rate can reach 78% in recurrent and refractory HLH, with low toxicity and good tolerability.^[29]^ Additionally, in adults with primary HLH, allo-HSCT achieves a 5-year overall survival rate of 73%.^[30]^ Notably, for SAA patients under 40 years of age with an HLA-matched sibling, allo-HSCT is considered the gold standard treatment.^[31]^ In this study, the patient was treated with dexamethasone combined with ruxolitinib for HLH, leading to a decrease in bilirubin levels and improvement in symptoms such as fatigue, though his fever persisted. The decision to initiate therapy with this combination rather than a standard etoposide regimen was made considering the dual burden of HLH and SAA-related pancytopenia. Etoposide was deemed too myelosuppressive, risking complete hematopoietic failure and jeopardizing the potential for a curative transplant. With a matched sibling donor identified, the immediate therapeutic goal became controlling the hyperinflammatory state to act as a bridge to allo-HSCT. Ruxolitinib, which is a second-line treatment option for HLH and can mitigate the HLH-associated cytokine storm with a more favorable toxicity profile, was selected as a bridging strategy for this purpose. The family’s concerns about the potential toxicity of combination chemotherapy were also integral to the treatment decision. Although the patient’s response was partial, it sufficed to proceed with the transplant. The decision to proceed with urgent allo-HSCT despite ongoing fever was based on a critical risk-benefit analysis. First, the patient’s severe pancytopenia from SAA was irreversible and the root cause of his vulnerability to infections. Neither the SAA nor the refractory HLH was likely to be controlled without restoring normal hematopoiesis. Second, allo-HSCT represented the only potential cure for both the bone marrow failure and the aberrant immune activation driving HLH, by replacing the dysfunctional immune system. Third, waiting for complete fever resolution was unlikely, as it was driven by the underlying immune dysregulation that only a transplant could correct. Ultimately, this strategy proved successful. Engraftment and immune reconstitution post-transplant led to the resolution of both the cytopenias and the hyperinflammatory state, confirming that allo-HSCT was the pivotal intervention for this case of HLH-complicated SAA.

In summary, this case underscores the considerable challenge in diagnosing and treating HLH complicating SAA. Our experience indicates that a high index of suspicion for HLH is warranted in SAA patients with persistent fever and hyperbilirubinemia. The central therapeutic imperative is to address both the bone marrow failure and the hyperinflammatory state. In this case, proceeding with allo-HSCT proved pivotal, as it aimed to correct the underlying immune dysregulation common to both conditions. Given the profound limitations inherent in a single case report, including the lack of a control group and the unique clinical course, our findings should be interpreted with caution, and definitive conclusions cannot be drawn. Therefore, the generalizability of our findings, as well as the efficacy of allo-HSCT for this high-risk combination, requires validation through future collaborative studies with larger cohorts.

## Author contributions

**Conceptualization:** Ming Jiang.

**Data curation:** Hailong Yuan.

**Formal analysis:** Jianli Xu, Hailong Yuan.

**Investigation:** Jianli Xu, Hailong Yuan, Gang Chen.

**Project administration:** Ming Jiang.

**Resources:** Jianli Xu, Hailong Yuan, Gang Chen, Jianhua Qu, Huan Wang, Rong Chen, Ming Jiang.

**Supervision:** Jianli Xu.

**Writing – original draft:** Jianli Xu.

**Writing – review & editing:** Jianli Xu, Hailong Yuan, Gang Chen, Jianhua Qu, Huan Wang, Rong Chen, Ming Jiang.
